# Histological Analysis of Extracranial Carotid Artery Aneurysms

**DOI:** 10.1371/journal.pone.0117915

**Published:** 2015-01-30

**Authors:** Janna C. Welleweerd, Bastiaan G. L. Nelissen, Dave Koole, Jean-Paul P. M. de Vries, Frans L. Moll, Gerard Pasterkamp, Aryan Vink, Gert Jan de Borst

**Affiliations:** 1 Vascular Surgery, University Medical Center Utrecht, Heidelberglaan 100, 3584CX Utrecht, the Netherlands; 2 Experimental Cardiology laboratory, University Medical Center Utrecht, Heidelberglaan 100, 3584CX Utrecht, the Netherlands; 3 Vascular Surgery, St Antonius Hospital, Koekoekslaan 1, 3435CM Nieuwegein, the Netherlands; 4 Pathology, University Medical Center Utrecht, Heidelberglaan 100, 3584CX Utrecht, the Netherlands; University of Amsterdam Academic Medical Center, NETHERLANDS

## Abstract

**Introduction:**

Extracranial carotid artery aneurysms (ECAA) are rare but may be accompanied with significant morbidity. Previous studies mostly focused on diagnostic imaging and treatment. In contrast, the pathophysiological mechanisms and natural course of ECAA are largely unknown. Understanding the pathophysiological background may add to prediction of risk for adverse outcome and need for surgical exclusion. The aim of this study was to investigate the histopathological characteristics of ECAA in patients who underwent complete surgical ECAA resection.

**Material and Methods:**

From March 2004 till June 2013, 13 patients were treated with open ECAA repair. During surgery the aneurysm sac was resected and processed for standardized histological analysis. Sections were stained with routine hematoxylin and eosin and special stains to detect elastin, collagen, different types of inflammatory cells, vascular smooth muscle cells and endothelial cells.

**Results:**

Histopathological characterization revealed two distinct categories: dissection (abrupt interruption of the media; n = 3) and degeneration (general loss of elastin fibers in the media; n = 10). In the degenerative samples the elastin fibers in the media were fragmented and were partly absent. Inflammatory cells were observed in the vessel wall of the aneurysms.

**Conclusion:**

Histological analysis in this small sample size revealed dissection and degeneration as the two distinct underlying mechanisms in ECAA formation.

## Introduction

Extracranial carotid artery aneurysms (ECAA) are rare with an incidence varying from 0.09 to 2.0% of all carotid surgical procedures.[1,2] Aneurysms of the extracranial carotid artery are defined as a dilatation of 50% or more of the diameter of the expected healthy carotid artery.[3] Studies on ECAA mostly comprise case reports or small case series focusing on diagnostic imaging and treatment outcome.[2,4–6] Although the natural course of ECAA is largely unknown, the clinical presentation of ECAA may be accompanied with significant morbidity. Previous studies reported a stroke prevalence of 50% and a mortality of 60–70% when ECAA is left untreated.[7]

Some authors suggest that small asymptomatic ECAA could be treated conservatively with strict follow-up, but surgery is generally the accepted treatment for symptomatic ECAA.[7–10] The etiology of ECAA is heterogeneous and includes atherosclerosis, post-dissection, trauma and infection.[2,11] The exact pathophysiological mechanisms however remain unclear, and prognostic factors for clinical outcome are largely unknown.[12] Detailed understanding of the mechanisms of ECAA and general aneurysm formation could be the start of improving diagnostics and treatment.

Accordingly, the present study was conducted to investigate the histopathological characteristics of ECAA in patients who underwent complete surgical ECAA resection.

## Methods

### Subjects

**Fig 1:**
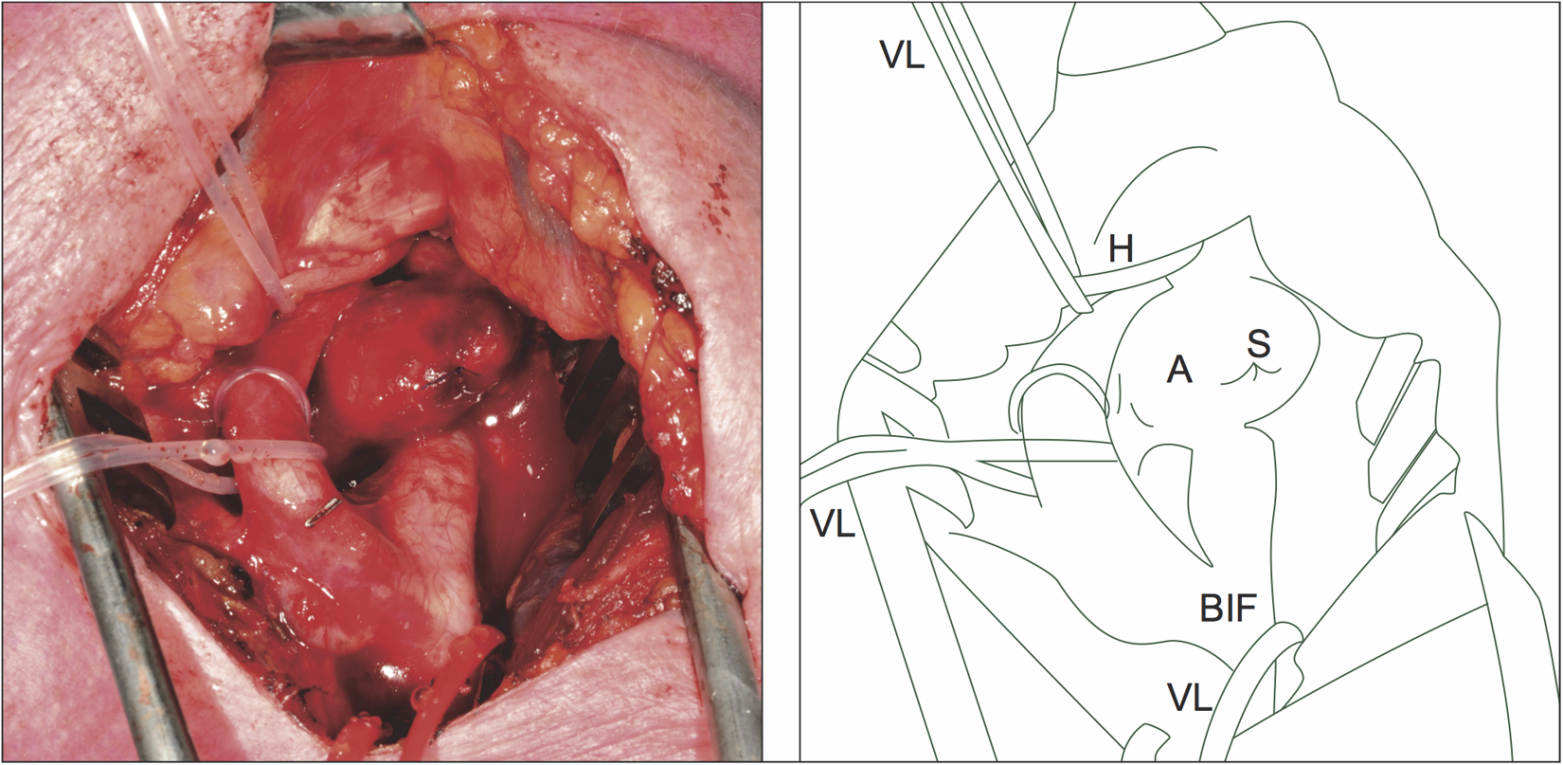
In vivo aneurysm. Aneurysm of a left saccular carotid artery visible between the internal carotid artery (ICA) and the external carotid artery (ECA) and originating from a dorsal loop in the ICA. The common carotid artery is ligatured in red, the ECA is identified with transparent ligatures. A, aneurysm of the ICA; BIF, carotid bifurcation; H, nervus hypoglossus; S, suture; VL, vessel loop.

From March 2004 till June 2013, 38 patients were treated for ECAA in the two participating hospitals (University Medical Center Utrecht, Utrecht, and St Antonius hospital Nieuwegein, the Netherlands). In this study, all patients (n = 15) that underwent open ECAA repair with complete aneurysm sac resection (Fig. 1) were included. Histological analysis was not possible in two patients due to incomplete preserved samples. Therefore, these two cases were excluded, leaving 13 cases for analysis. Operation indication was decided on after multidisciplinary deliberation and based on presenting symptoms, location, and ECAA size. The medical ethics committees of both participating hospitals (Verenigde Commissies Mensgebonden Onderzoek, St Antonius hospital Nieuwegein and Medisch Ethische Toetsings commissie University Medical Center Utrecht) approved the study and all study participants provided written informed consent.

### Imaging

Morphological characteristics of the aneurysms were assessed by preoperative imaging diagnostics. Computed tomography angiogram (CTA) was used in ten patients, magnetic resonance angiography (MRA) was used in two, and conventional angiography in one patient.

### Sampling

During open surgical repair, the complete ECAA sac was collected and subsequently processed for histological analysis. The specimen was fixed in 4% formaldehyde, decalcified for 1 week in ethylenediaminetetraacetic acid and embedded in paraffin. Of the paraffin segments, 4-μm-thick sections were cut for histological analyses. Sections were stained for elastin (elastin von Gieson), collagen (Sirius red), macrophages (CD68), T-lymphocytes (CD3), B-lymphocytes (CD 20), plasma cells (CD 138), endothelial cells (CD34) and vascular smooth muscle cells (SMC) (alpha-smooth muscle actin). In addition a routine hematoxylin and eosin staining was performed.

### Result interpretation

Elastin was graded as an estimation of the percentage of media containing elastin fibers. Collagen was graded as an estimation of the percentage present in the vessel wall. The presence of inflammatory cells in aneurysm wall was semi-quantitatively scored as minor or heavy staining. Dissection was defined as an abrupt interruption of the media with signs of organized thrombus in the tear of the vessel wall. Degeneration was defined as decrease of elastin in the media. Histological examination was retrospectively performed collectively by three independent observers (BN, DK and AV) unaware of clinical data. In case of discrepancies in judgment, sections were reanalyzed until consensus was reached.

### Controls

Post-mortem non-aneurysmal carotid specimens from five patients with a median age of 63 (range 51–90) without relevant medical history were used as controls. Two bodies were donated for education and research to University Medical Center Utrecht. Written and witnessed consent for body donation was given prior to death by both controls. Three more specimens were collected in patients for whom the carotid artery was investigated for diagnostic reasons in an autopsy procedure. The use of these specimens is described in the code of proper use of human tissue that is used in the Netherlands.[13]

### Statistical analysis

Discrete variables are shown as frequencies and percentages of the total. Continuous variables are shown as median and interquartile range. Categorical variables were investigated using the chi-square test or the Fisher’s exact test. Continuous variables were compared using the Student’s t-test. P value ≤. 05 was considered statistically significant.

## Results

### Clinical patients characteristics

Thirteen patients with a median age of 55 years (IQR: 35–75, six males) were included. Baseline characteristics are presented in Table 1. All treated patients were symptomatic and the most common symptom was cerebral ischemia (N = 6) (see Table 2). All but one of these patients had ischemia due to (temporary) occlusion of the ipsilateral medial cerebral artery. One patient presented with ischemia of the contralateral medial cerebral artery and a controlateral Horner syndrome.

**Table 1:** Patient characteristics.

	Total (n = 13)
Gender male	6 (46%)
Age, years ^a^	55 (35–75)
PAD	1 (8%)
Hypertension	7 (54%)
MI	1 (8%)
COPD	1 (8%)
Hypercholesterolemia	2 (15%)
Connective tissue disorder	0 (0%)
Smoking	4 (33%)
DM	0 (0%)
Statin use	6 (46%)

Data are presented as No. (%) unless otherwise indicated.

^a^ Median and interquartile range (IQR). Abbreviations. PAD, peripheral artery disease; MI, myocardial infarction; COPD, chronic obstructive pulmonary disease; DM, diabetes mellitus.

**Table 2:** Aneurysm characteristics.

Gender/age	Location	Size(mm)	Morphology	Symptoms	Pathology
M/76	LICA	30	Fusiform	Stroke	Degenerative
F/65	RICA	NR	Fusiform	Pain, mass	Degenerative
F/41	LICA	4	Saccular	TIA/Stroke	Dissection
M/53	RICA	34	Saccular	Pulsatile mass	Degenerative
M/46	RCCA	13	Fusiform	Pain, mass	Degenerative
M/55	LICA	NR	Fusiform	TIA	Dissection
M/50	LICA	12	Fusiform	TIA	Degenerative
M/26	LICA	46	Fusiform	Pain, mass	Degenerative
M/47	RICA	5	Saccular	TIA	Dissection
F/66	RICA	15	Saccular	Pain	Degenerative
F/75	LICA	27	Fusiform	CL TIA, Horner	Degenerative
F/62	LICA	12	Saccular	Hoarseness	Degenerative
F/67	LICA	27	Saccular	Mass	Degenerative

Abbreviations. M male; F female; LICA left internal carotid artery; RICA right internal carotid artery; RCCA right common carotid artery; NR not reported; TIA transient ischemic attack; CL contralateral.

### Imaging findings

ECAAs were mostly located in the Internal carotid artery (ICA) (n = 12) and one ECAA was located in the common carotid artery (CCA). Eight aneurysms were located on the left side. Seven aneurysms were fusiform and six were saccular. The aneurysms varied in size; the median length was 30mm (range: 10–100) and the mean outer diameter 13mm (range: 4–46). There were no radiological signs of dissection pre-operative in any of the aneurysms. Thrombus was present in two aneurysms and six aneurysms had calcifications in the vascular wall. Presence of thrombus or calcifications was not significantly different in degenerative aneurysms or aneurysms after dissection (p = .164 and p = .577).

### Controls

**Fig 2:**
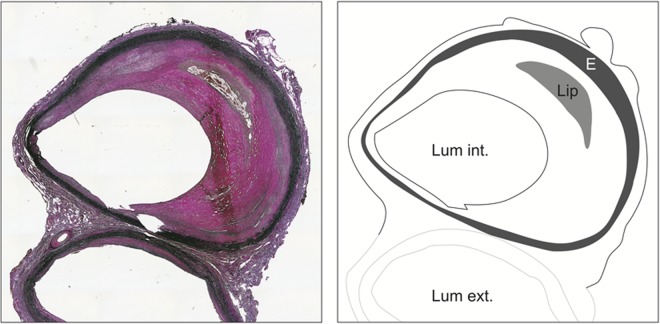
Histology of control sample: fibrous cap atheroma. Histology of control sample. Sample taken just distal from the bifurcation. Elastin-van Giesson (EvG) stain. In black the elastic fibers are clearly present and well organized. Atherosclerotic changes, atheroma with a lipid core. E, Elastin; Lip, Lipid core; Lum ext., lumen of the external carotid artery; Lum int., lumen of the internal carotid artery.

Our five control ICA samples showed no media degeneration or dissection. In all samples the elastin, smooth muscle cells, and collagen fibers were present and well organized (see Table 3). Inflammatory cells were absent in all but one control specimens. Two samples did show atherosclerotic lesions (sample 3 and 5) that could be classified as a fibrous cap atheroma and pathological intimal thickening (Modified American Heart Association Classification) (Fig. 2).[14] The atheroma was surrounded by CD68 positive macrophages.

**Table 3:** Histology characteristics.

		Control (n = 5)	Dissection (n = 3)	Degeneration (n = 10)	P value
Elastin^a^		95% (30–100%)	25% (2–50%)	30% (0–95%)	.063
Smooth muscle^a^		88% (15–100%)	95% (60–100%)	65% (5–95%)	.004
Vasa vasorum	minor	4 (80%)	1 (33%)	3 (30%)	1.000
	heavy	1 (20%)	2 (67%)	7 (70%)	
Collagen	minor	3(60%)	2 (100%)	1 (10%)	.014
	heavy	2 (40%)	0 (0%)	9 (90%)	
Lymphocytes	minor	5 (100%)	3 (100%)	6 (60%)	.497
	heavy	0 (0%)	0 (0%)	4 (40%)	
Plasma cells	minor	5 (100%)	3 (100%)	10 (100%)	NA
	heavy	0 (0%)	0 (0%)	0 (0%)	
B-Lymphocyte	minor	5 (100%)	3 (100%)	9 (90%)	1.000
	heavy	0 (0%)	0 (0%)	1 (10%)	
T-lymphocytes	minor	5 (100%)	3 (100%)	6 (60%)	.497
	heavy	0 (0%)	0 (0%)	4 (40%)	
Macrophages	minor	4 (80%)	3 (100%)	4 (40%)	.192
	heavy	1 (20%)	0 (0%)	6 (60%)	

Continuous data are presented as median (range). Categoric data are presented as number (%) of heavy staining as opposed to minor staining unless otherwise indicated.

^a^ Percentage of fibers present in a non-diseased vessel wall.

### Samples

**Fig 3:**
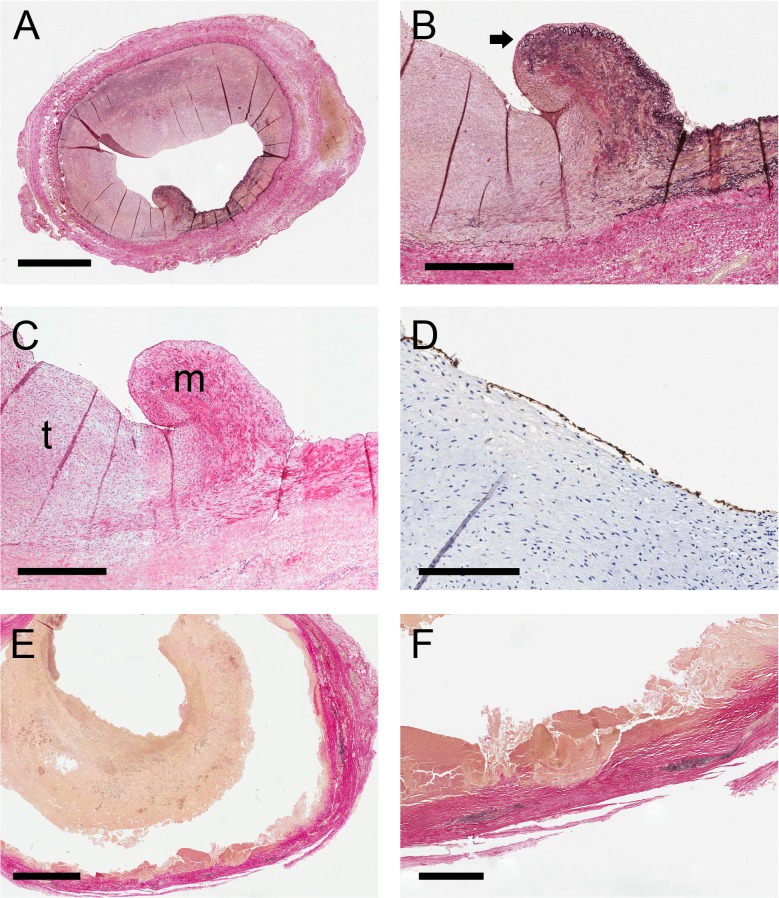
Histology of carotid aneurysms. A-D, dissection; E and F, degeneration. A, overview of aneurysm due to dissection. Elastin-van Giesson (EvG) stain. Bar = 1.5 mm. B, higher magnification of the same staining as A. Arrow indicates the disrupted internal elastic lamina. Bar = 500 μm. C, Hematoxylin and eosin staining of the same panel as B. m, media; t, organized thrombus that replaces the absent media. Bar = 500 μm. D, CD34 immunostain showing endothelial coverage of the thrombus (in brown). Bar = 250 μm. E, overview of an aneurysm due to degeneration. Elastin-van Giesson (EvG) stain. Bar = 4 mm. F, higher magnification of the same staining as E. In black remnants of the elastic fibers of the media. Bar = 1 mm.

Histological examination was retrospectively performed, four sections were reanalyzed because of discrepancies in judgment between the independent observers, in all sections consensus was reached. Histological examination revealed two distinct categories of ECAA (Table 3). The majority (n = 10; 77%) of the samples showed a distinct degenerative pattern of the vessel wall without signs of a dissection (Fig. 3). In the degenerative samples the elastin fibers of the media were fragmented and were partly absent (Fig. 3E-3F). Inflammatory cells were present in each sample. In most degenerative samples inflammatory cells were clearly present, both lymphocytes and macrophages were seen; only three samples scored ‘minor’ for all observed types of inflammatory cells.

The remaining three samples (23%) showed a dissection with an abrupt interruption of the medial layer (Fig. 3A-3D). The gap in the arterial wall was filled with an organized thrombus with groups of myofibroblasts. All dissective samples showed marks of degeneration in the media and scored ‘minor’ for the different inflammatory cells in the vessel wall. There were no significant differences in any type of inflammatory cell between samples with dissection or samples with degeneration (Table 3). We did find significant differences in SMC and collagen. SMC was higher in the dissection group and collagen was higher in the degenerative samples.

## Discussion

In the current study, histological examination of ECAA showed two distinctive categories: aneurysms after dissection versus degenerative aneurysms. Retrospectively, there were no radiological signs of dissection pre-operative in any of the aneurysms.

Clinically and radiological diagnosed aneurysmal formation after previous dissection has been described in literature.[15] In the aorta and its branches, degeneration is a well-recognized cause for aneurysm formation.[16,17] Non-dissective causes of peripheral aneurysms, such as carotid or popliteal aneurysms, are believed to exist but had not been histologically confirmed yet.[18] We found degenerative aneurysms in 10/13 (73%) patients. The fact that we observed inflammatory cells in the aneurysms suggest that inflammation might also play a role.

It must be pointed out that the possibility exists that these two distinctive categories could be different stages of the same disease. Although decrease of SMC was higher in the degenerative samples, degeneration of the medial layer was also observed in all dissection cases and could eventually weaken the vascular wall and facilitate dissection of the intimal and medial layer. Increase of collagen in aneurysms has been demonstrated in analysis of other aneurysmal vessels.[19] Theoretically, while collagen is load-bearing at large dimensions and elastin being load-bearing at small dimensions the collagen-elastin ratio changes in aneurysms.[20]

The result of this study could be the start of understanding the mechanism or mechanisms of ECAA development. This could eventually lead to improved treatment strategies. For example, in most cases of dissecting aneurysms invasive treatment is not indicated, as a major part of these aneurysms remains asymptomatic and does not increase in size while some even spontaneously resolve.[21] On the contrary, atherosclerotic origin of ECAA may need more aggressive intervention, although there is a clear need for further natural follow-up in patients with ECAA in general.[12]

There are some study limitations. Most important, it must be noticed that this study was performed on a small group of patients, consequently our findings have little power and definitive conclusions are difficult to draw. This might also explain why we did not find any aneurysms with another etiology, for example mycotic aneurysms, in our analysis. However, ECAA is a rare disease and only around 1000 cases have been reported in the international literature so far.[12] Furthermore, only a selected subgroup of patients presenting with ECAA will undergo surgical resection of the aneurysm. As a consequence, also in our tertiary referral center, the number of patients operated on is limited and therefore it is hard to study larger patient groups. Second, in this study patients were selected based on intervention. The result of this study and the conclusions drawn from them only apply to patients that underwent surgical repair for their ECAA.

In conclusion, this is the first histological study of samples taken from ECAA. Histopathological ECAA characterization in this study revealed two distinct categories: dissection and degeneration. The result of this study could be used as a basis for understanding the mechanism of ECAA development. Further study in resected ECAA is needed to explore the clinical relevance of this mechanism.
